# Low-chloride versus high-chloride hypertonic solution for the treatment of subarachnoid hemorrhage-related complications (The ACETatE trial): study protocol for a pilot randomized controlled trial

**DOI:** 10.1186/s13063-018-3007-7

**Published:** 2018-11-14

**Authors:** Ofer Sadan, Owen Samuels, William H. Asbury, John J. Hanfelt, Kai Singbartl

**Affiliations:** 1 Department of Neurology and Neurosurgery, Division of Neurocritical Care, Emory University Hospital and Emory School of Medicine, 1364 Clifton Road NE, Atlanta, GA 30322 USA; 2 Department of Pharmacy, Emory University Hospital, 1364 Clifton Road NE, Atlanta, GA 30322 USA; 3 Department of Biostatistics and Bioinformatics, Emory University, 1518 Clifton Road NE, Atlanta, GA 30322 USA; 4 Department of Critical Care Medicine, Mayo Clinic, Phoenix, AZ 85054 USA

**Keywords:** Subarachnoid hemorrhage, Osmotherapy, Hypertonic NaCl, Acute kidney injury, Hyperchloremia

## Abstract

**Background:**

Aneurysmal subarachnoid hemorrhage (aSAH) is a life-threatening condition that results from a ruptured cerebral vessel. Cerebral edema and vasospasm are common complications and frequently require treatment with hypertonic solutions, in particular hypertonic sodium chloride (NaCl). We have previously shown that hyperchloremia in patients with aSAH given hypertonic NaCl is associated with the development of acute kidney injury (AKI), which leads to higher morbidity and mortality. Our current trial aims to study the effect of two hypertonic solutions with different chloride content on serum chloride concentrations in patients with aSAH who are at risk for AKI.

**Methods:**

A low ChloridE hyperTonic solution for brain Edema (ACETatE) is a single center, double-blinded, double-dummy pilot trial comparing bolus doses of 23.4% NaCl and 16.4% NaCl/Na-Acetate for the treatment of cerebral edema in patients with aSAH. All patients will be enrolled within 36 h following admission. Randomization will occur once patients who receive hypertonic treatment for cerebral edema develop hyperchloremia (serum Cl^−^ concentration ≥ 109 mmol/L). Subsequent treatment will consist of either NaCl 23.4% or NaCl/Na-Acetate 16.4%. The primary outcome of this study will be the change in serum Cl^−^ concentrations during treatment. Secondary outcomes will include incidence of AKI, mortality, changes in intracranial pressure, and extent of hypernatremia.

**Discussion:**

In patients with aSAH, hyperchloremia is a known risk factor for subsequent development of AKI. The primary goal of this pilot study is to determine the effect of two hypertonic solutions with different Cl^−^ content on serum Cl^−^ concentrations in patients with aSAH who have already developed hyperchloremia. Data will be collected prospectively to determine the extent to which the choice of hypertonic saline solution affects subsequent serum Cl^−^ concentrations and the occurrence of AKI. This approach will allow us to obtain preliminary data to design a large randomized trial assessing the effects of chloride-sparing hypertonic solutions on development of AKI in patients with SAH. This pilot study is the first to prospectively evaluate the relationship between hypertonic solution chloride content and its effect on serum electrolytes and renal function in aSAH patients at risk of AKI due to hyperchloremia.

**Trial registration:**

Clinicaltrials.gov, NCT03204955. Registered on 28 June 2017.

**Electronic supplementary material:**

The online version of this article (10.1186/s13063-018-3007-7) contains supplementary material, which is available to authorized users.

## Background

Aneurysmal subarachnoid hemorrhage (aSAH) is a life-threatening condition that results from a rupture in a cerebral artery [1]. Risk factors include female gender, hypertension, smoking, and excessive alcohol consumption [2]. The overall incidence is estimated at 5–10 per 100,000 per year [3]. Although treatment has improved in recent years, aSAH still represents a potentially devastating clinical condition, with a reported in-hospital mortality of approximately 20% [4]. The clinical course is often complicated by aneurysm re-bleeding, cerebral vasospasm with delayed cerebral ischemia, cerebral edema, and systemic complications, such as cardiopulmonary failure, electrolyte dysregulation, infections, and acute kidney injury (AKI). Systemic complications account for up to 40% of all deteriorations during inpatient management [5]. AKI in aSAH patients is associated with a greater than four-fold increase in hospital mortality, along with a less favorable neurological recovery in survivors [6, 7].

Hyperosmolar therapy is a cornerstone in the treatment of aSAH-related cerebral edema, which occurs in approximately 20 to 30% of this patient population [8]. Osmotherapy is based on elevation of serum osmolality via hypernatremia (e.g., hypertonic NaCl) or the use of an osmotically active agent (e.g., mannitol) [9]. The goal of osmotherapy is to reduce intracranial pressure and improve cerebral blood flow by raising serum osmolarity and subsequently inducing excess fluid shifts from brain tissue into the blood stream. Hypertonic sodium chloride (NaCl) solutions, 3% to 23.4%, are commonly used to induce hypernatremia, but carry the risk of significant hyperchloremia.

Intravenous chloride load has been reported to cause negative effects on blood pressure, kidney blood flow, and fluid retention in animals [10] and, subsequently, in healthy human subjects [11, 12]. In a variety of critically ill patient populations, hyperchloremia has also been observed to directly correlate with AKI and mortality [13, 14]. Furthermore, comparative studies have noted an association between isotonic NaCl infusions and an increased risk of AKI and poor outcome in critically ill patients [15, 16]. Summarizing 21 prospective studies, a meta-analysis by Krajewski et al. [17] demonstrated that high Cl^−^ containing solutions increase the risk of AKI, the number of blood transfusions, and the need for mechanical ventilation in perioperative or critically ill patients. Surprisingly, a multicenter trial failed to show a benefit of balanced intravenous (IV) solutions, i.e., low Cl^−^ solution, over high Cl^−^ solutions (NaCl 0.9%) in a mixed population of critically ill patients [18]. However, even more recent publications did demonstrate favorable renal outcomes in non-critically ill [19] and critically ill [20] patients receiving balanced crystalloid solution when compared with NaCl 0.9%. Since hypertonic NaCl solutions are not balanced solutions, it is not surprising to encounter controversy regarding their role in the development of AKI [21, 22].

In a recently published retrospective analysis of SAH patients, we observed correlations between hyperchloremia and AKI, and between AKI and increased morbidity and mortality. We identified treatment with hypertonic NaCl as a risk factor for the development of hyperchloremia [7]. Nonetheless, we were not able to show a dose-response relationship between chloride load and subsequent development of hyperchloremia [7]; instead we found great variability in chloride exposure. The large variability appeared to be related to a high incidence of cerebral salt wasting syndrome in our study population, resulting in the need for extensive sodium and volume replacement with IV fluids, especially NaCl solutions at various concentrations [23].

Therefore, the question arises whether avoiding further worsening of hyperchloremia by differential use of hypertonic solutions might be a better strategy to prevent AKI in critically ill patients (with SAH) than simply replacing high Cl^−^ solutions with low Cl^−^ solutions in all cases.

**Fig. 1:**
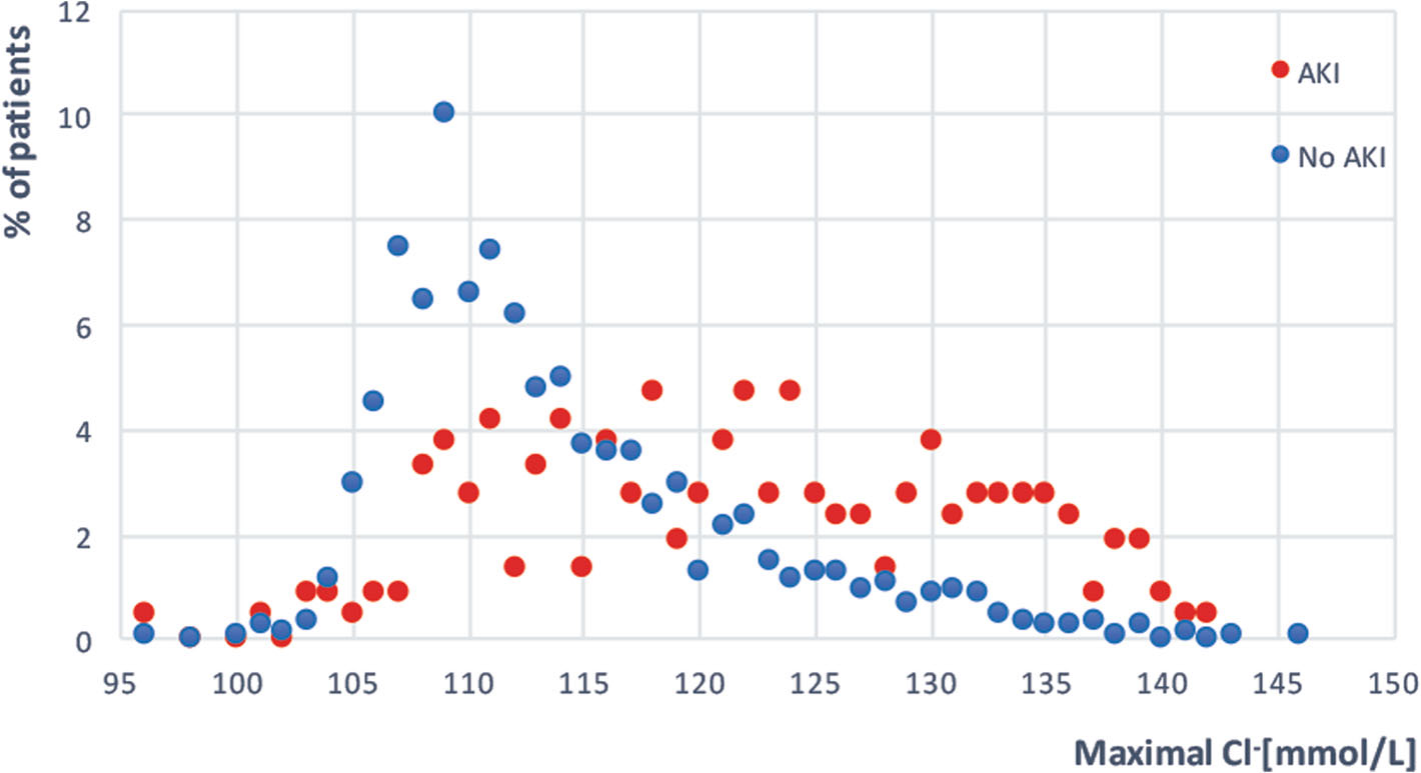
Distribution of maximal chloride serum measurements between acute kidney injury (AKI) and non-AKI as a percent of patients in a retrospective cohort of SAH patients

Additional analyses of our recent study further support this approach [7]. The frequency of maximum chloride levels in patients without AKI increased up to a chloride concentration of 109 mmol/L and decreased thereafter (Fig. 1). On the contrary, the frequency of maximum Cl^−^ levels increased and remained elevated for serum Cl^−^ levels greater than 109 mmol/L in patients with AKI (Fig. 1). Moreover, only 8.5% of all AKI patients had maximum serum Cl^−^ concentrations < 109 mmol/L. By setting a threshold of 109 mmol/L, we would therefore exclude only a small proportion of AKI patients. Moreover, patients without hyperchloremia appear unlikely to benefit from preventive measures with low Cl^−^ solutions. Focusing on at-risk patients should allow us to reduce variability and consequently provide more stringent answers in this highly controversial area of AKI research.

To this end, our proposed trial described below intends to make use of the different Cl^−^ content in NaCl 23.4% or NaCl/Na-Acetate 16.4% (Table 1) and thereby to examine the causative role of hypertonic IV solutions in the development of hyperchloremia (and subsequently AKI) in patients with SAH. Our study design represents a double-blinded, double-dummy, pragmatic approach.

**Table 1:** Composition of the two hypertonic solutions compared in the trial

	NaCl (standard treatment) group	NaCl/Na-Acetate (alternate treatment) group
Solution components (per dose)	Sodium chloride	Sodium chloride and sodium acetate
Concentration (%)	23.4	16.4
NaCl 23.4% pre-mixed solution (mL)	30	20
Na-Acetate 3% pre-mixed solution (mL)	0	30
Volume (mL)	30	50
Sodium content (mEq/dose)	120	140
Chloride content (mEq/dose)	120	80
Acetate content (mEq/dose)	0	60

## Methods and study design

### Study objectives

In a single-center, double-blinded, double-dummy pilot design we will compare NaCl 23.4% and NaCl/Na-Acetate 16.4% (Table 1) for the treatment of cerebral edema in patients with aneurysmal SAH and evolving hyperchloremia (serum Cl^−^ ≥ 109 mmol/L).

The primary objective of the A low ChloridE hyperTonic solution for brain Edema (ACETatE) study will be to assess longitudinal chloride serum change in aSAH patients treated with either NaCl 23.4% (30 mL) or NaCl/Na-Acetate 16.4% (50 mL). Secondary outcomes will be: the rate of new-onset AKI according to KDIGO criteria [24]; all causes in-hospital mortality, including withdrawal of treatment or discharge to a hospice facility; and confirmation of a comparable effect of NaCl/Na-Acetate 16.4% and NaCl 23.4% for treatment of intracerebral hypertension. The latter will be assessed by: 1) effect on intracerebral pressure (ICP) when treatment indication is for elevated ICP; and 2) response in serum sodium concentration following administration.

### Study settings

This will be a single-center trial in a major referral neurocritical care unit with more than 200 new admissions for SAH of any cause each year. All new aSAH admissions to the Neurocritical Care Unit at Emory University Hospital will be screened for eligibility by the intensive care unit (ICU) team upon arrival. Our experience, as well as data from the literature, suggest that non-aneurysmal SAH patients (e.g., idiopathic or “angio-negative”) often have a more benign clinical course than aneurysmal SAH patients in terms of cerebral edema, vasospasm, and ultimately the need for osmotherapy [25, 26]. We will enroll only aneurysmal SAH patients to minimize heterogeneity. Consent will be obtained by the neurocritical care team affiliated with the study.

### Study design

This is a prospective, double-blind, double-dummy pilot clinical trial. Patients will be enrolled within the first 36 h from admission to the ICU. Inclusion criteria include:

- Spontaneous SAH with an identified aneurysmal source as noted on neuroimaging.
- Age ≥ 18 years

Exclusion criteria include:

- SAH related to non-aneurysmal etiology—e.g., other vascular source, trauma, negative workup (“angio-negative”), etc.
- Patients who arrive in a brain-death state or in a devastating clinical status that will likely to lead to brain death or early withdrawal from curative treatment
- Patients who suffer from end-stage renal disease at baseline or who are currently receiving renal replacement therapy
- Known pregnancy

**Fig. 2:**
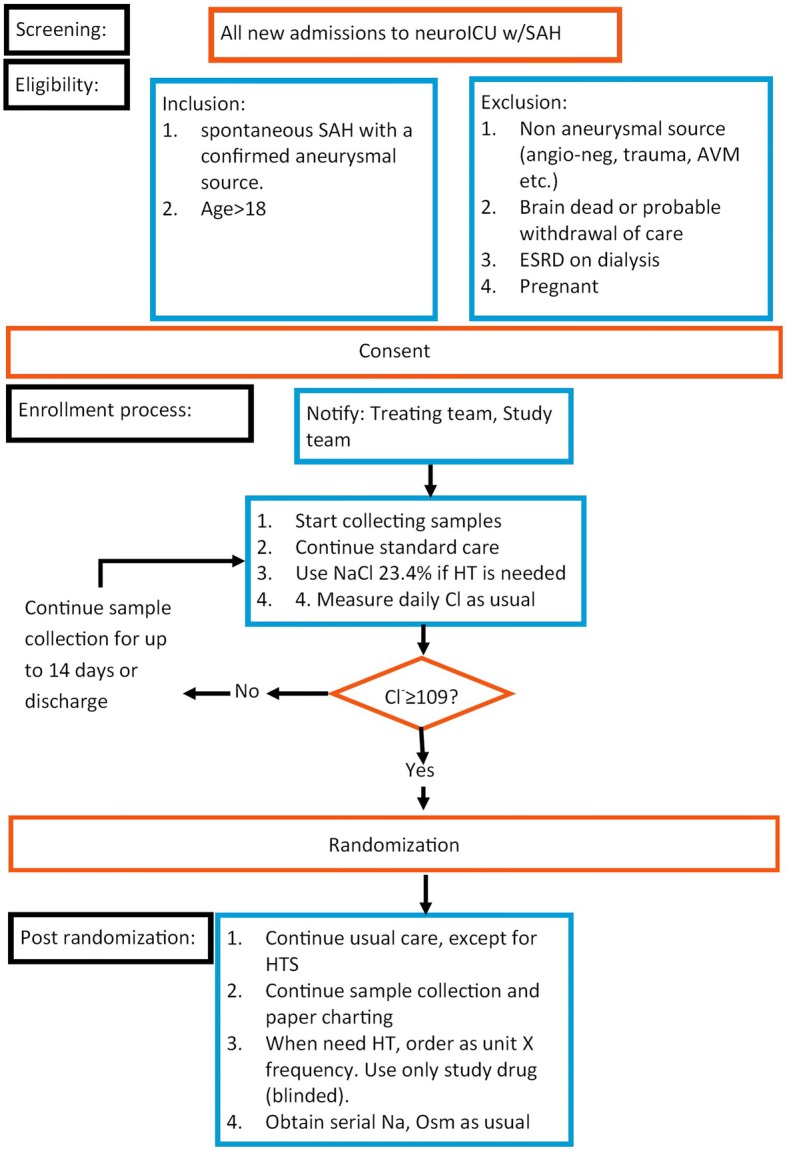
ACETatE study flowchart. AVM arteriovenous malformation, ESRD end-stage renal disease, HT hypertonic, HTS hypertonic sailne, ICU intensive care unit, SAH subarachnoid hemorrhage

**Fig. 3:**
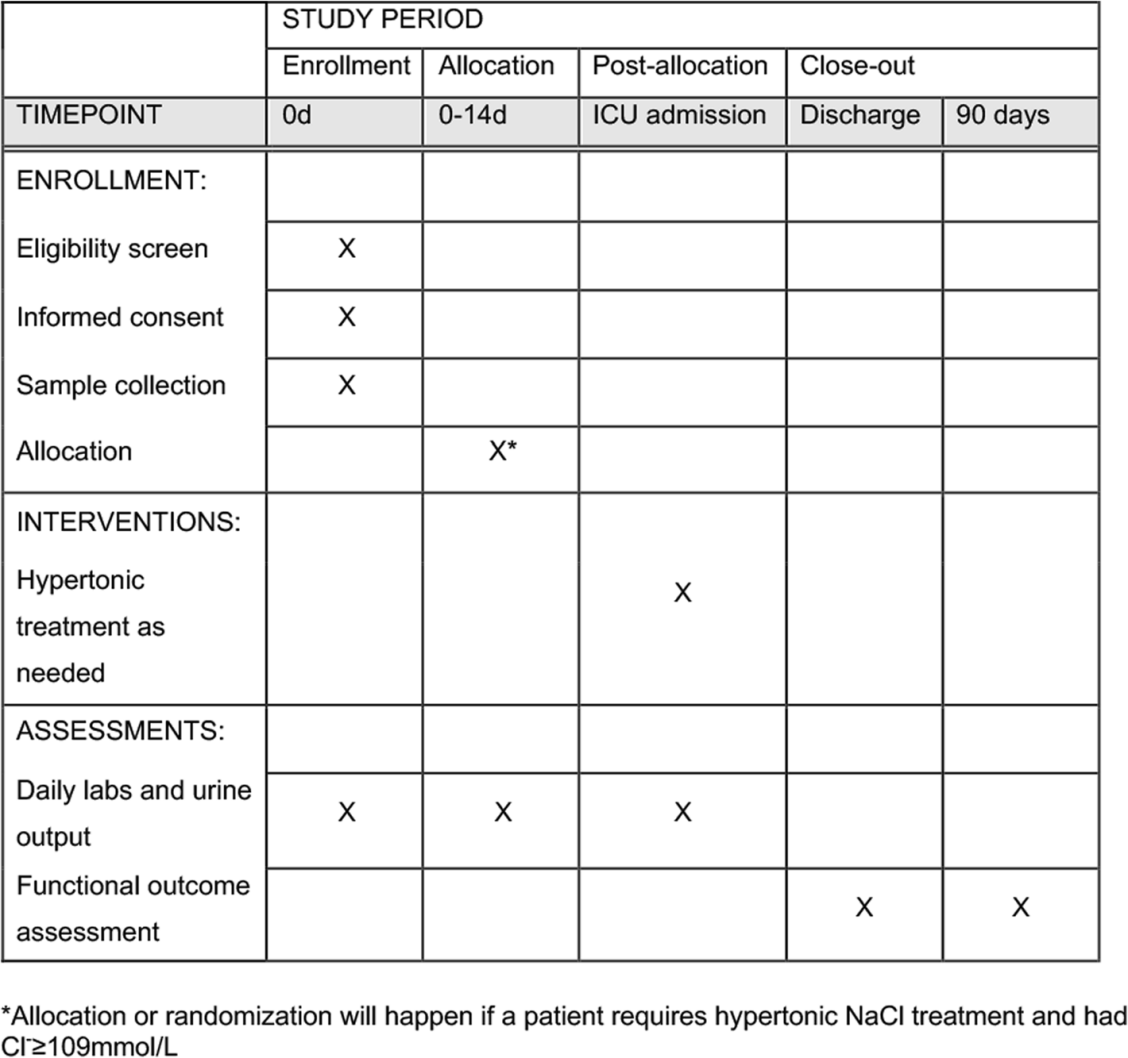
Schedule of enrollment, interventions, and assessments. ICU intensive care unit

Figures 2 and 3 outline the study flow chart and schedule, respectively. A Standard Protocl Items: Recommandations for Clinical Interventional Trials (SPIRIT) checklist is also available (see Additional file 1).  Patients will be eligible for enrollment up to 36 h after ICU admission, a time period during which the etiology of the SAH (aneurysmal versus non-aneurysmal) is usually validated, and surgical or endovascular treatment is completed.

All patients will initially receive standard SAH care [27]. Commencement and dose of hypertonic treatment (NaCl 23.4%) for cerebral edema will be at the discretion of the attending physician. Serum Cl^−^ levels will be monitored daily for all patients. Patients will become eligible for randomization if their serum Cl^−^ reaches 109 mmol/L or above. Actual randomization will occur only when the attending physician decides to continue with hypertonic treatment.

For randomization to NaCl or the NaCl/Na-Acetate group (1:1 ratio), we will use random block sizes and will stratify by: 1) serum creatinine level on admission (normal vs. abnormal according to our clinical laboratory normal range); and 2) SAH Hunt and Hess score, i.e. low (1–2) vs. high (3–5) [28]. Randomization will be conducted centrally, under Emory’s investigational drug service responsibility, to avoid potential unblinding of group allocation of the clinical team, who will not be able to access the allocation list until the end of the trial, specifically until the 90-day status is obtained for the last patient enrolled. In case the attending physician decides that the blinded treatment does not achieve its goals, an unblinded treatment will be initiated instead.

As the hypertonic NaCl and NaCl/Na-Acetate study solutions are not equal in volume, we have developed a double-blinded, double-dummy design in which all patients will receive the treatment dose in one bag and a second bag containing the placebo solution. Patients will blindly receive either: 1) 30 mL NaCl 23.4% IV piggyback (ivpb) plus a 50-mL infusion of dummy solution (balanced electrolyte solution: Plasma-Lyte A) ivpb over 20 min; or 2) 50 mL NaCl/Na-Acetate16.4% ivpb plus a 30-mL ivpb infusion of a dummy solution over 20 min. There will be no other difference in the treatment of the patients. Both bags will be administered concurrently via separate ports of a central line.

After consent is obtained, patient demographic and clinical information will be collected. Study data will be stored and managed using REDCap, a HIPPA compatible secure database, hosted at Emory University [29]. Patient identity on REDCap will be coded to ensure patient anonymity. For each randomized patient that receives the study drug, we will use a specific charting aid to document medication administration and adverse events once per shift. A study safety and data monitoring committee has been established to review trial data once a year or upon recruiting half of the planned patients, whichever comes first, which consists of neurocritical care attendings not related to the trial and a biostatistician. The committee will evaluate differences in in-patient mortality as the main indication to terminate the trial, and will assess serious reported adverse effects.

We further plan to collect and store urine and serum samples daily as well as cerebrospinal fluid (CSF) samples, if available, up to three times a week. These samples will be stored for future analysis and will not be part of the primary study analysis. Following patient discharge, we will assess their survival, disposition (e.g., home, long-term acute care, etc.) and functional capacity (measured by modified Rankin scale) at approximately 90 days post-bleed, either during a planned outpatient visit or by phone.

### Sample size and statistical analysis

Based on our retrospective analysis previously mentioned [7], we anticipate a maximal change in serum Cl^−^ of 10 mmol/L for patients randomized to the 30-mL 23.4% NaCl group. In patients randomized to the 50-mL 16.4% NaCl/Na-Acetate group, we hypothesize that a maximal change in serum Cl^−^ of 4 mmol/L will occur. By randomizing 60 patients for this pilot trial (30 patients per group), we will have a 63% power to detect a six-point difference in the change in serum Cl^−^ levels (4 mmol/L vs. 10 mmol/L) between treatment groups (two-sided test, type I error rate 0.05). The data analysis will follow the intent-to-treat principle.

To randomize 60 eligible patients with serum Cl^−^ levels of 109 mmol/L or greater who require hypertonic treatment, we will need to consent a higher number of patients. In our cohort analysis [7], only 15% of all patients did not develop serum Cl^−^ levels ≥ 109 mmol/L. Therefore, we will consent up to 100 patients and randomize the first 60 that meet our eligibility requirements.

## Discussion

We propose a double-blind, double-dummy, prospective clinical trial to compare the effect of two hypertonic saline solutions with different Cl^−^ content on serum Cl^−^ levels in patients with cerebral edema due to aSAH. Our immediate goal is to collect high-quality prospective data regarding the adverse effects, effectiveness, and feasibility of our current hypertonic solution dosing strategies. In the future, we plan to design a larger randomized trial.

This is the first trial to prospectively assess the relationship between the Cl^−^ content of two hypertonic solutions with different Cl^−^ content and their effect on serum electrolytes as well as renal function.

The question whether balanced IV fluid solutions, i.e., low Cl^−^ solutions, improve patient outcome when compared with isotonic NaCl has been controversial for several years [30, 31]. Although some studies have demonstrated associations between higher IV chloride content and AKI, recent prospective studies have produced conflicting results [18–20, 32]. Failure to focus on specific patient populations, i.e., patients at risk for AKI, could be one reason for these inconsistent results.

Hypertonic solutions are, by definition, non-balanced and represent an extreme case of IV Cl^−^ exposure. In a recent retrospective study, we have demonstrated a relationship between hyperchloremia, the use of hypertonic solutions, and the development of AKI in patients with SAH. Unfortunately, our data did not reveal a dose-response relationship between IV Cl^−^ exposure and AKI [7]. However, our findings have allowed us to identify a group of SAH patients who are at risk for AKI. As only 8.5% of our patients with maximal serum Cl^−^ concentrations < 109 mmol/L developed AKI, we consider serum Cl^−^ concentrations ≥ 109 mmol/L as a risk factor for AKI in patients with SAH, and propose a corresponding study design. So far, no clinical trial has based commencement of randomized treatment on the evolution or presence of a specific risk factor, such as hyperchloremia, when studying the effects of IV solutions with different Cl^−^ content.

The ACETatE trial will allow for a direct assessment of the dose-response relationship between the IV solution and serum concentrations, for both sodium and chloride. We are following a pragmatic trial design, as there is no clear, widely accepted protocol for use of hypertonic solutions in patients with SAH. Dose and frequency of osmotherapy will be at the discretion of the attending physician. The treatment for study patients will be nearly identical to that of any other patient with SAH in our ICU. Treatment will only differ by the Cl^−^ content of the hypertonic solution to which the patients will be randomized.

An important, potential limitation of our study is the comparison of two solutions with different volumes and Na^+^ concentrations. Although the total Na^+^ load will be lower in the more concentrated solution, the precise effects of 30 ml of 23.4% Na^+^ solution versus those of 50 ml of 16.4% Na^+^ solutions on serum osmolarity, serum sodium, and ICP remain unknown.

Another potential pitfall is insufficient enrollment due to the limitations of the informed consent process in critically ill patients [33]. First, patients are often unable to consent themselves due to the cerebral injury while a next of kin cannot be contacted in time. Second, patients or their families have great difficulty consenting for a trial given the seriousness of the illness and deciding on it within a relatively short period of time.

Even if we achieve our goal of patients, our power to detect the needed difference is only 63%. These calculations have dictated the primary goal of preventing extreme hyperchloremia, and not AKI, which is too rare to change in a small, single-center trial.

However, the ACETatE trial, with all the mentioned limitations and possible confounding circumstances, will follow the changes in serum electrolyte concentration as a function of IV exposure. This high-quality information will help us to design a large-scale clinical trial which is desperately needed in the debate about the optimal Cl^−^ content in the IV solution [30, 31].

## Trial status

This trial has been approved by the Emory University IRB (protocol version #4, dated 6/8/2017, clinicaltrials.gov #NCT03204955). Recruitment is ongoing.

## Additional file

Additional file 1: SPIRIT 2013 checklist: recommended items to address in a clinical trial protocol and related documents. (DOC 122 kb)

## Abbreviations

AKI: Acute kidney injury
CSF: Cerebrospinal fluid
ICP: Intracerebral pressure
IRB: Institutional Review Board
SAH: Subarachnoid hemorrhage
